# Antimicrobial and anticancerogenic activity of selenium nanoparticles obtained via pulsed laser ablation

**DOI:** 10.1038/s41598-026-44730-y

**Published:** 2026-05-05

**Authors:** Entidhar Jasim Khamees, Lubna Abdulazeem, Zaytoon Abdulrida Al-Khafaji, Olcay Gençyılmaz

**Affiliations:** 1 Department of Physiology and Medical Physics, Babylon University, Hilla, Iraq; 2 DNA-Research Center, University of Babylon, Hilla, Iraq; 3 Department of Microbiology, College of Medicine, University of Babylon, Hilla, Iraq; 4 Department of Physics, Çankırı Karatekin University, Çankırı, Turkey

**Keywords:** Selenium nanoparticles, Antibacterial-anticancer CaCo-2 MTT assay, Laser ablation, Chemical reduction, Biochemistry, Biotechnology, Cancer, Chemistry, Drug discovery, Microbiology

## Abstract

SeNPs were synthesized via chemical reduction and physical pulsed laser ablation techniques. Stable, spherical nanoparticles with TEM diameters ranging from 20 to 150 nm (mean ± SD: 28.5 ± 7.2 nm and 33.8 ± 6.4 nm for the chemical and physical methods, respectively) were obtained. The characteristic absorption peaks at 310 nm and 371 nm were detected for SeNPs produced by chemical reduction and physical pulsed laser ablation, respectively. The band gap values were found to be 1.74 eV and 1.92 eV, respectively. Zeta potential analysis confirmed moderate colloidal stability (23.67 mV and 25.17 mV for chemical and physical reduction, respectively). The antibacterial activities of the SeNPs against *E. coli*, *Klebsiella spp.*, *Pseudomonas spp.* and *Staphylococcus spp.* were evaluated using the agar well diffusion method. SeNPs synthesized by the chemical reduction method formed inhibition zones of 10, 14, 18, 20 and 27 mm (*E. coli*); 9–18 mm (*Staphylococcus spp.*); 10–17 mm (*Klebsiella spp.*); and 8–16 mm (Pseudomonas spp.). Particles synthesized by physical pulsed laser ablation formed equivalent zones of 9–24 mm, 8–16 mm, 9–17 mm, and 7–15 mm. A particularly notable result was the strain- and dose-dependent difference identified by one-way ANOVA (F = 3304.3, *p* < 1 × 10⁻^12^) at 1000 μg/mL for chemical SeNPs. Anticancer activity was evaluated against CaCo-2 colon adenocarcinoma cells using the MTT assay. Chemically synthesized SeNPs were found to reduce viability from 90 ± 4.5% (6.25 μg/mL) to 18 ± 1.1% (200 μg/mL) with an IC₅₀ of approximately 57 μg/mL (R^2^ = 0.996). Physically prepared SeNPs were found to have viability ranging from 91.8% to 33.3%, with an IC₅₀ value of approximately 36 μg/mL. The Pearson correlation between log [dose] and viability was found to be highly negative (r = − 0.979 to − 0.992, *p* < 0.001). Overall, SeNPs exhibited dose-dependent antibacterial activity (> 27 mm inhibition) and anticancer activity (IC₅₀ ≤ 57 μg/mL), with the synthesis route affecting efficacy. These quantitative observations confirm the SeNPs’ potential as multifunctional nanotherapeutics, necessitating in vivo studies to definitively determine pharmacokinetics (PK), safety, and mechanistic pathways.

## Introduction

The development of effective capabilities against bacteria and CRC is an increasingly urgent issue due to the rising incidence of multidrug resistance (MDR) in bacteria and the growing global burden of CRC. Classic antibiotics are ineffective against emerging bacterial strains, and the use of current chemotherapy is limited by systemic toxicity, drug resistance and a lack of selectivity against cancer cells^1,2^. Nanotechnology has had a significant impact on modern medicine through the production of materials such as nanoparticles (NPs), which have unique properties such as high surface reactivity and tunable size. Targeted studies and production using these nanomaterials are becoming increasingly functional^3^. Selenium nanoparticles (SeNPs) are the focus of extensive research due to their strong bioactivity, low toxicity and biocompatibility. Selenium is an important trace element with antioxidant properties^4^, and when designed as a nanoparticle it is a promising antimicrobial, anticancer, anti-inflammatory and immunomodulatory agent^4^. From an antibacterial point of view, the antimicrobial activity of SeNPs is attributed to several pathways, such as:

- Perturbation of the bacterial cell wall and membrane structure.
- Induction of ROS-mediated oxidative stress.
- Inhibition of bacterial DNA replication and metabolism^5,6^.

These features render SeNPs a potential alternative to antibiotics, especially against resistant species such as *Klebsiella* and *Pseudomonas*. Conversely, SeNPs have shown outstanding potential in oncology research, particularly in the targeted delivery of drugs to cancer cells. Studies using CaCo-2 colorectal adenocarcinoma cells have demonstrated SeNPs’ ability to cause mitochondrial disruption, caspase-dependent apoptosis and dose-dependent cell death^7^. SeNPs can also enhance the therapeutic effects of other drugs or act as carriers for targeted treatment^8^. The aim of this study is to integrate the antibacterial, antifungal and anticancer assessment of SeNPs into a comprehensive biological profile of their activity.

This study focused on SeNPs, which are trace elements that have rarely been studied in this field. The SeNPs were synthesized using two methods: chemical reduction and physical laser ablation. The physical, antibacterial and anticancer properties of the resulting SeNPs synthesised using these methods were investigated. The antibacterial effect of the SeNPs on pathogenic bacteria was examined using an agar culture medium containing *Escherichia co*li, *Pseudomonas spp.*, *Staphylococcus spp.* and *Klebsiella spp.*, and the inhibition zones were analyzed using vitro models to support the in vivo data. The cytotoxicity of SeNPs against CaCo-2 cancer cells was determined using the MTT assay at various concentrations of the nanoparticles. A comparative study was conducted on bacteria and human cancer cells treated with SeNPs to evaluate the therapeutic window, dose-dependent efficacy and potential biomedical applications. This dual evaluation strategy provided researchers with a broader perspective.

## Methods and materials

### Synthesis of selenium nanoparticles via laser ablation

**Fig. 1:**
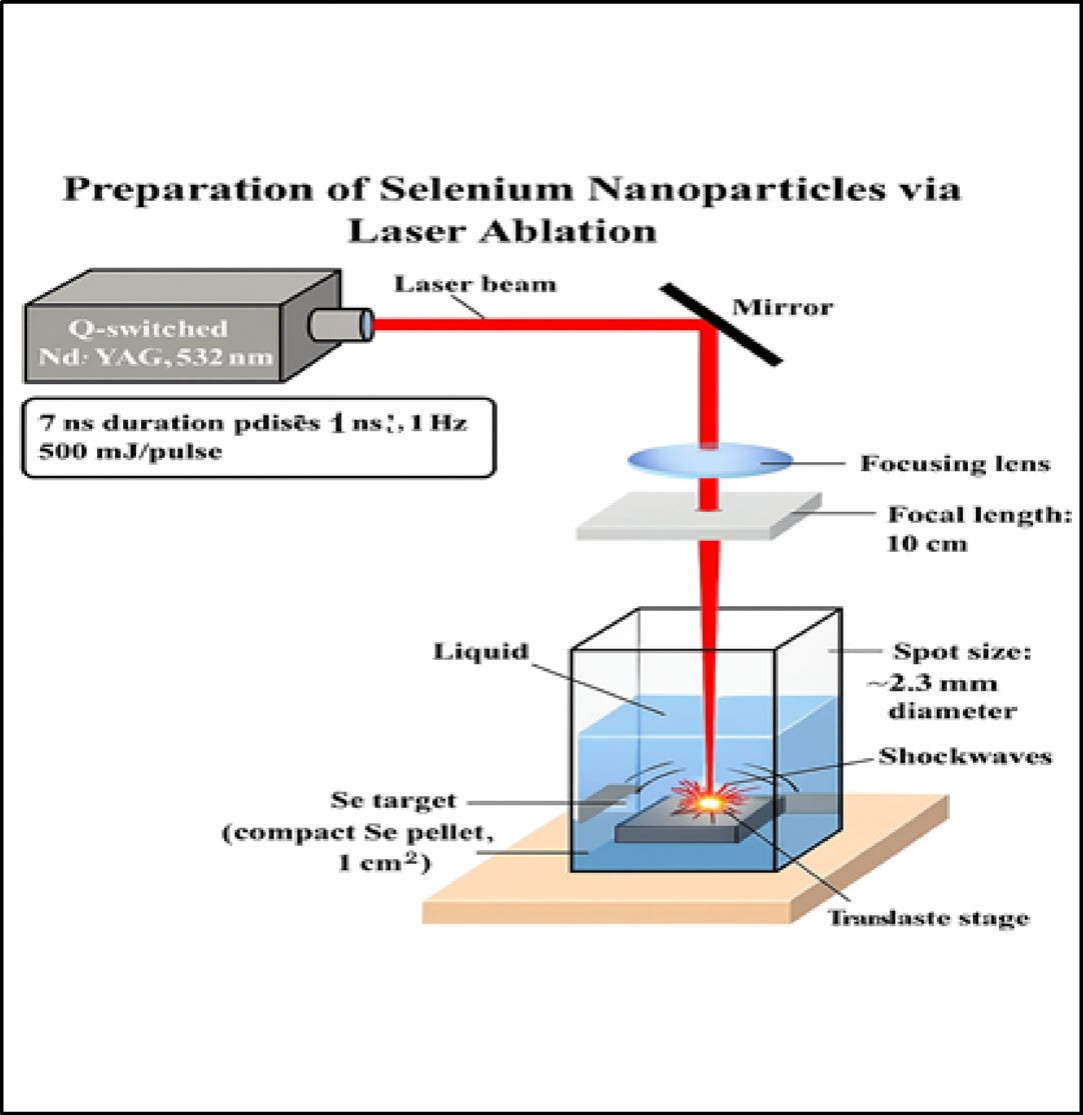
The schematic representation of the synthesis of SeNPs by laser ablation in distilled water.

Selenium nanoparticles (SeNPs) were synthesized using laser ablation in liquid (LAL), a clean and eco-friendly method that avoids the use of chemical reducing agents^9^. A 1 cm^2^ selenium (Se) pellet was submerged in 5 ml of distilled water in a borosilicate glass vessel at room temperature. The selenium target was placed at the bottom of the vessel to ensure stable irradiation conditions. A Q-switched Nd:YAG laser (HUAFEI, China), operating at a wavelength of 532 nm with a pulse duration of 7 ns and a repetition rate of 1 Hz, was used for ablation. The laser energy was set at 500 mJ per pulse. A converging lens with a focal length of 10 cm was used to focus the laser beam onto the surface of the selenium target, producing a spot size of approximately 2.3 mm in diameter^10^. As the number of laser pulses increased during the ablation process, the solution color changed from colorless to reddish-brown, indicating the formation of colloidal Se NPs^11^. The laser ablation in liquid technique allows precise control over particle size and purity, making it particularly suitable for biomedical applications^12^ (Fig. 1).

### Synthesis SeNPs by chemical reduction

**Fig. 2:**
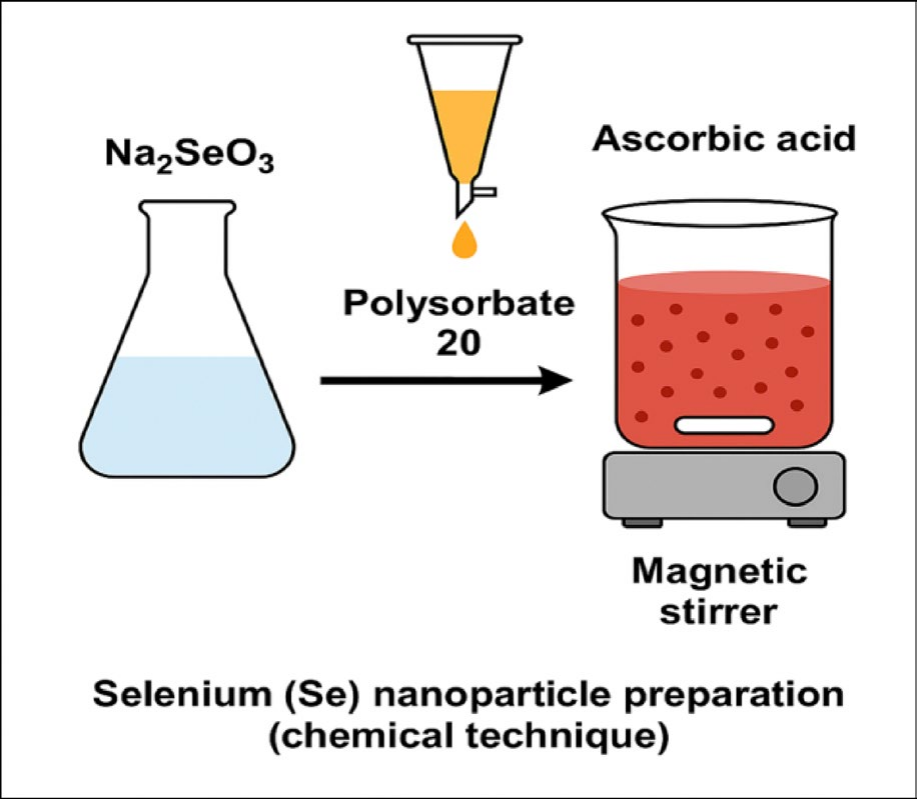
The schematic representation of the synthesis of SeNPs by chemical reduction.

Selenium nanoparticles (SeNPs) were prepared using chemical reduction methods, whereby a reducing agent facilitates the conversion of selenium ions into elemental selenium, which is then stabilized to prevent agglomeration. Sodium selenite (Na_2_SeO_3_.5H_2_O) was used as the selenium precursor and ascorbic acid (vitamin C) as the reducing agent due to its excellent electron-donating properties and biocompatibility. Furthermore, polysorbate 20 was used as a stabilizing agent to maintain the colloidal stability of the synthesized nanoparticles. Briefly, 30 mg of Na_2_SeO_3_ was dissolved in 90 mL of milli-Q water containing a magnetic stir bar. Then, an ascorbic acid solution (56.7 mM, 10 mL) was added dropwise to the above solution at room temperature with vigorous agitation. The color of the solution gradually changed as the selenite ions were reduced and colloidal SeNPs were produced. This process can easily be scaled up and has low toxicity, making it suitable for biomedical applications^13–15^. Ascorbic acid plays a role in creating a mild, non-toxic reaction environment and surfactants such as polysorbate 20 prevent particle aggregation and improve dispersion stability^16^ (Fig. 2).

### Cell lines and culture

The Caco-2 human colorectal cancer cell line was obtained from the National Cell Bank of Iran (Pasteur Institute, Tehran). The cells were cultured in RPMI-1640 medium (Gibco, USA), supplemented with 10% foetal bovine serum (FBS; Gibco, USA) and antibiotics (100 U/mL penicillin and 100 μg/mL streptomycin). The cells were cultured at 37 °C in a humidified atmosphere containing 5% CO₂. Subculturing was performed using 1 × trypsin/EDTA (Gibco, USA) and phosphate-buffered saline (PBS). Ethical approval was not sought as this was a small-scale experimental study of a well-established, commercially available human cancer cell line and neither human participants nor primary human tissue were involved.

### MTT cell viability assay

Cell viability was determined using the MTT (3-(4.5-dimethylthiazol-2-yl)-2.5-diphenyltetrazolium bromide) assay (Sigma-Aldrich, USA). The cells were detached using trypsin/EDTA, harvested and seeded into 96-well plates at a density of 1.4 × 10^4^ cells per well in a volume of 200 μL of complete culture medium. Following a 24-h incubation period to allow cell attachment, the cells were treated with various concentrations (200–12.5 μg/mL) of the tested compounds, which were dissolved in DMSO. The cells were then incubated for an additional 24 h at 37 °C in 5% CO₂. After treatment, the culture medium was replaced with 200 μL of an MTT solution (0.5 mg/mL in PBS). The plates were then incubated for a further 4 h at 37 °C to allow formazan crystal formation. The supernatant was then gently removed, after which 100 μL of dimethyl sulfoxide (DMSO) was added to each well to dissolve the crystals. Absorbance was read at 570 nm using a microplate reader (Wave XS2, BioTek, USA). Cell viability was presented as a percentage of the untreated control cells. Half-maximal inhibitory concentration (IC₅₀) values were then calculated from the corresponding dose–response curves.

### Characterization of SeNPs

The as-prepared selenium nanoparticles (SeNPs) were synthesized and characterized using UV–visible (UV–Vis) spectroscopy and scanning electron microscopy (SEM) to confirm their formation and physicochemical properties. UV–Vis spectroscopy was used to identify the surface plasmon resonance (SPR) band, with absorbance spectra collected from 330 to 660 nm. The presence of a unique absorbance peak within this range is characteristic of SeNP generation, resulting from the collective oscillation of electrons on the surface of the nanoparticle. The evolution of the precursor solution was also supported by a visual color change, e.g. from pale yellow to red–orange, which indicates the formation of nanoparticles^17,18^.

The morphology and particle size distribution were determined by SEM. The size and morphology images revealed predominantly spherical SeNPs and showed that the mean diameter varied from 20 to 150 nm under different synthesis methodologies. To ensure excellent uptake within the cell and biological activity, these nanoparticles should be in the nanoscale range^19,20^. Taken together, these methods enable the synthesis of stable, defined selenium NPs that are suitable for bioapplications. This study provides a direct comparison of chemically reduced and laser-ablated SeNPs within the same experimental framework, emphasizing their dual antibacterial and anticancer functions and linking their optical, structural and biological properties.

## Results and discussion

### Structural analysis

X-ray diffraction (XRD) was used to carry out the crystallographic analysis of SeNPs prepared through laser ablation and chemical reduction techniques. This approach is critical for evaluating the crystallinity, purity, and structural orientation of nanomaterials. Both SeNP samples exhibited sharp, well-defined diffraction peaks at 2θ positions of 23.5°, 29.2°, 41.4°, 43.3°, 45.4°, 52.5°, 55.7° and 62.7°. These peaks correspond to the crystallographic planes (100), (101), (110), (102), (111), (201), (112) and (202). These planes correspond to the hexagonal wurtzite phase of selenium, as reported in JCPDS card No. 06-0362^16^. The XRD diffraction pattern of the laser-prepared SeNPs was characterized by sharper, more intense peaks, particularly near the (101) plane. This demonstrates the nanoscale’s high crystallinity and well-defined structure. In contrast, chemically synthesized SeNPs revealed somewhat broader and less intense peaks, which are usually associated with smaller crystallite sizes or less order.

**Fig. 3:**
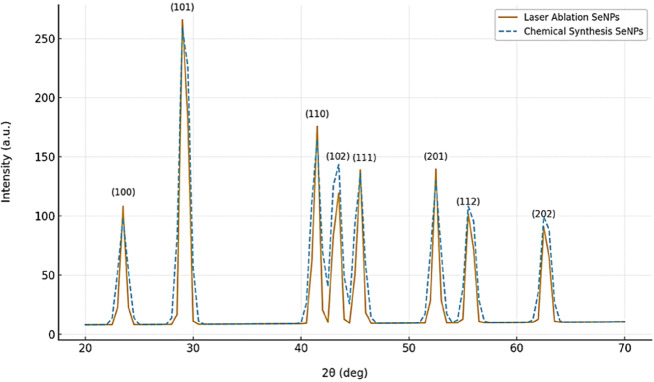
The X-ray diffraction patterns of SeNPs.

Crucially, no extra peaks besides the selenium nanoparticle peak were observed using either technique, indicating the high purity of the selenium nanoparticles and the absence of secondary phases or impurities. It was also observed that differences in the power of the laser pulse did not affect the crystallinity of the laser-synthesized SeNPs, thus demonstrating the robustness of this method for producing nanostructures. This corroborates previous reports discussing the advantages of laser ablation for achieving high-purity crystalline nanoparticles, although chemical synthesis remains a more easily scalable process (Fig. 3).

### UV–Vis analysis

**Fig. 4:**
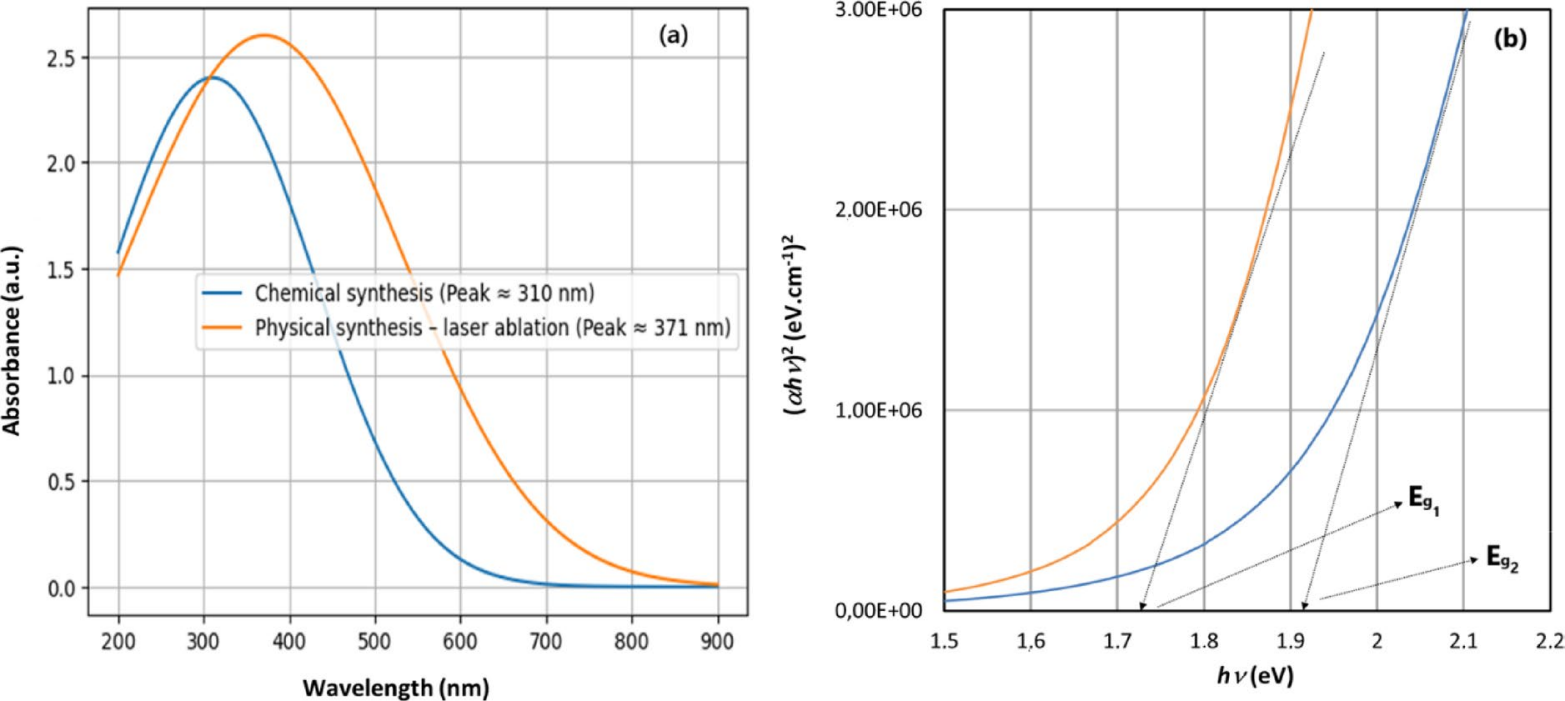
(**a**) The absorption spectra and (**b**) Tauc plots of SeNPs.

UV–Vis absorption spectra were taken to characterize the absorption onset and assess synthesis-dependent optical performance of synthetic selenium nanoparticles (SeNPs) prepared by chemical reduction and physical laser ablation over wavelengths from 200 to 900 nm (Fig. 4a). The chemically synthesized SeNPs exhibited a distinctive absorption peak at approximately 310 nm following treatment, resulting from the encapsulation of organic stabilizing agents (e.g. ascorbic acid or polysorbate) on the surface. These agents are also useful for improving nanoparticle stability, dispersibility, and bio-system interaction^21,22^. In comparison, SeNPs produced by pulsed laser ablation exhibited a significant red shift in the absorption maximum at 371 nm, which is typically associated with surface plasmon resonance (SPR). This red-shifted signature indicates that the optical response of nanoparticles with absent or lower levels of organic surface capping is more intrinsic to their size and electronic structure than that of nanoparticles synthesized chemically^23,24^.

Tauc plots were used to determine the optical band gap (*E*_*g1*_* and E*_*g2*_), providing a clearer picture of the optical characteristics (see Fig. 4b). The band gap of the chemically synthesized SeNPs was calculated to be relatively large (*E*_*g2*_ = 1.92 eV), likely due to the combined effects of quantum confinement and surface passivation facilitated by organic molecules^25^. By contrast, laser-ablated SeNPs displayed a significantly lower band gap of *E*_*g1*_ = 1.74 eV, indicating a more delocalized electronic structure and improved charge carrier mobility^26^. The differences in UV–Vis absorption values and band gap values were highly consistent with variability in particle size, as verified by dynamic light scattering (DLS) analysis. Laser-ablated SeNPs exhibited larger average particle sizes and smaller band gaps, promoting more effective electron transfer and increased reactive oxygen species (ROS) generation^27,28^. This optical–structural correlation may explain the superior antibacterial activity of physically synthesized SeNPs compared to chemically synthesized SeNPs. Overall, these studies reveal that the synthesis pathway determines the optical band structures of selenium nanoparticles, which consequently have a direct effect on their biological and biomedical function^29^.

### Zeta potential analysis

**Fig. 5:**
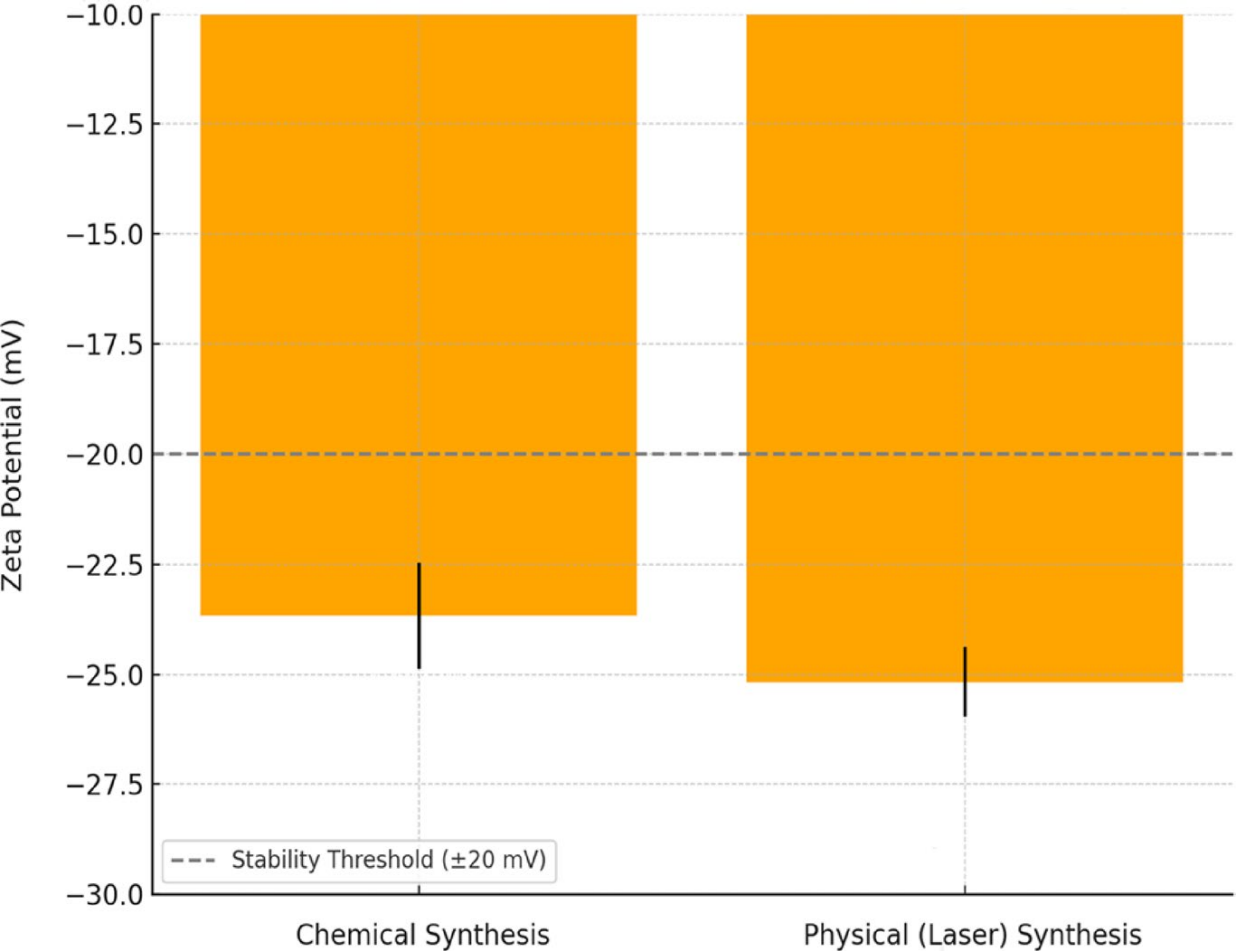
Zeta potential distribution of SeNPs.

**Fig. 6:**
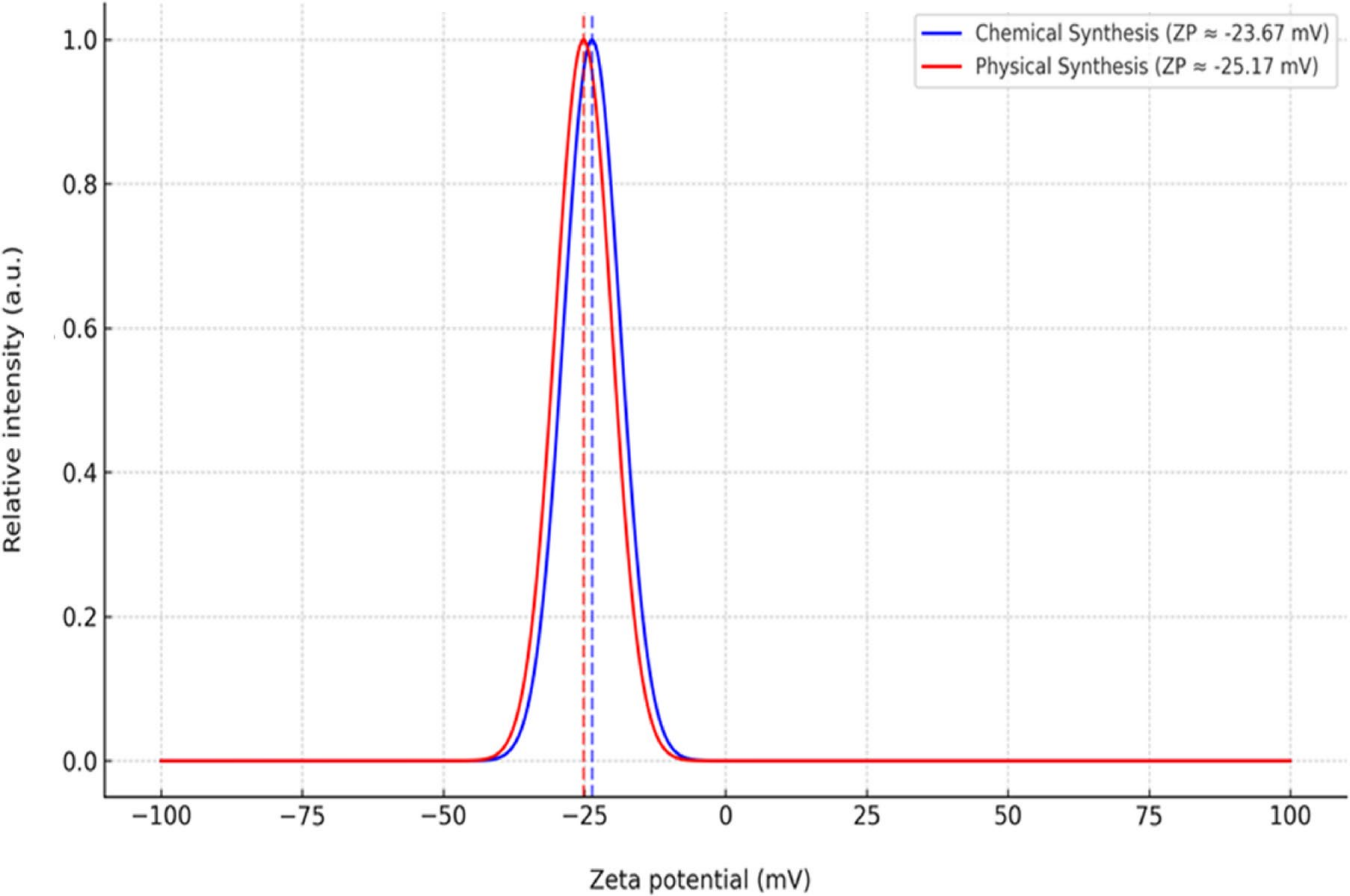
Comparative zeta potential distributions for SeNPs.

Surface charge and colloidal stability analyses were performed to evaluate the physicochemical properties of the SeNPs. The results of these analyses are presented in Figs. 5 and 6. Zeta potential measurements are an important parameter for determining the stability of nanoparticles in a colloidal environment. The zeta potential of SeNPs produced by chemical synthesis was found to be − 23.67 mV, while that of SeNPs produced by physical (laser) synthesis was found to be − 25.17 mV. According to the literature, absolute zeta potential values of ± 20 mV and above provide sufficient electrostatic repulsive forces between nanoparticles to support colloidal stability. In this context, it can be concluded that the SeNPs produced by both synthesis methods are colloidally stable.

**Fig. 7:**
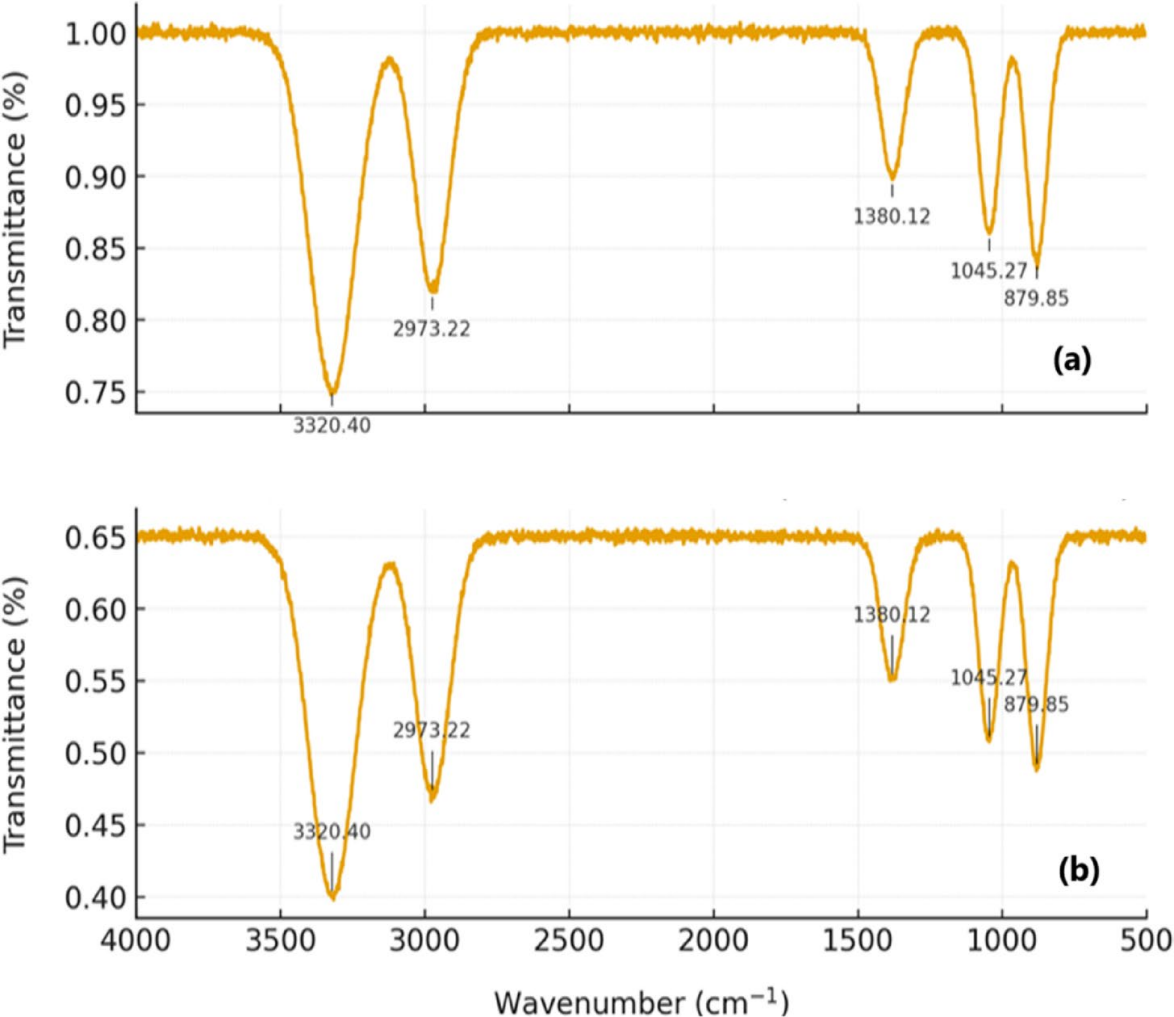
FTIR spectra of SeNPs synthesized by (**a**) pulsed laser ablation and (**b**) chemical reduction.

The SeNPs obtained by physical synthesis have a more negative zeta potential, which indicates stronger electrostatic repulsive forces between the nanoparticles. This can help the colloidal system to remain stable for longer by reducing the tendency of SeNPs to agglomerate. The absence of chemical reduction agents in physical production methods, such as laser ablation, may enable the SeNPs’ surface to remain cleaner and more homogeneous. This results in a more consistent surface charge distribution and enhanced electro kinetic properties. Examining the zeta potential distribution curves reveals that both samples exhibit a narrow, single-peak (monomodal) distribution. This indicates homogeneous surface charge properties of the SeNP population and that the synthesis processes occurred in a controlled manner^28^. The higher negative ZP of the physically prepared SeNPs indicates that these particles are more stable colloidally. This can be explained by their functionalized clean surface, free from stabilizers (unlike the chemically manufactured nanoparticles, which can have adsorbed organic molecules (e.g., ascorbate, polysorbate). These compounds have the potential to modulate the charge distribution of the surface^29^. These differences can have profound effect on the biological interactions such as drugs/cells internalization53, aggregation behavior54 and antimicrobial action54. The simulated relative plot as shown in Figs. 6 and 7, in addition, also emphasizes the narrowness (full width at half maximum = 2 ZP ), as well as the symmetry of the intensity distribution wrt its central ZP value. The blue curve corresponds to chemically synthesized SeNP, the red one is irradiated ablated SeNP. The narrow width and high peak symmetry in both samples are indicative of the homogenous distribution of particles. Particularly, the laser-ablated SeNPs show a slightly sharper profile, agreeing with the more homogeneous charge distribution in the surfactant-free systems^30,31^. Furthermore, the narrow distribution profile reveals that there are no multiple populations of nanoparticles with different surface properties in the system, and that the SeNPs exhibit similar electro kinetic behavior in the colloidal medium. Such homogeneous distributions enable SeNPs to exhibit more predictable behavior in sensitive applications for biomedical application.

### FTIR analysis

Figure 7 shows the FTIR spectrum of selenium nanoparticles (SeNPs). The surface chemistry and functional groups evident in the FTIR spectra indicate that SeNP aggregation is important for nanoparticle formation and stabilization. Both spectra show distinct broad peaks at values of ∼3320.96 cm⁻^1^ and ∼2973.22 cm⁻^1^, which are primarily attributed to O–H stretching vibrations, indicating the presence of hydroxyl groups from natural coating materials such as lignin, polysaccharides, or alcohols. These peaks confirm that these functional groups play a common role in SeNP stabilization. Additionally, the absorption bands at 1380.12–1045.27 cm⁻^1^ are attributed to the vibrations of carboxyl groups (–COOH) and C–O stretching; these vibrations can trigger electrostatic or hydrogen bond interactions on the NP surface. The additional peaks at approximately 1030 cm⁻^1^ and 879.65 cm⁻^1^ also indicate the presence of phenolic –OH groups or substituted aromatic rings, which could be responsible for the reduction and stabilization of the nanoparticles. In contrast, the local SERS spectra of SeNPs (red spectrum) are sharper and more resolved, particularly in the fingerprint region (< 1500 cm⁻^1^), indicating that laser ablation produces cleaner substrates with less organic contamination. In contrast, chemically prepared SeNPs exhibit broad and low-resolution peaks, which may indicate the presence of a wider range of heterologous organic compounds.

### SEM analysis

**Fig. 8:**
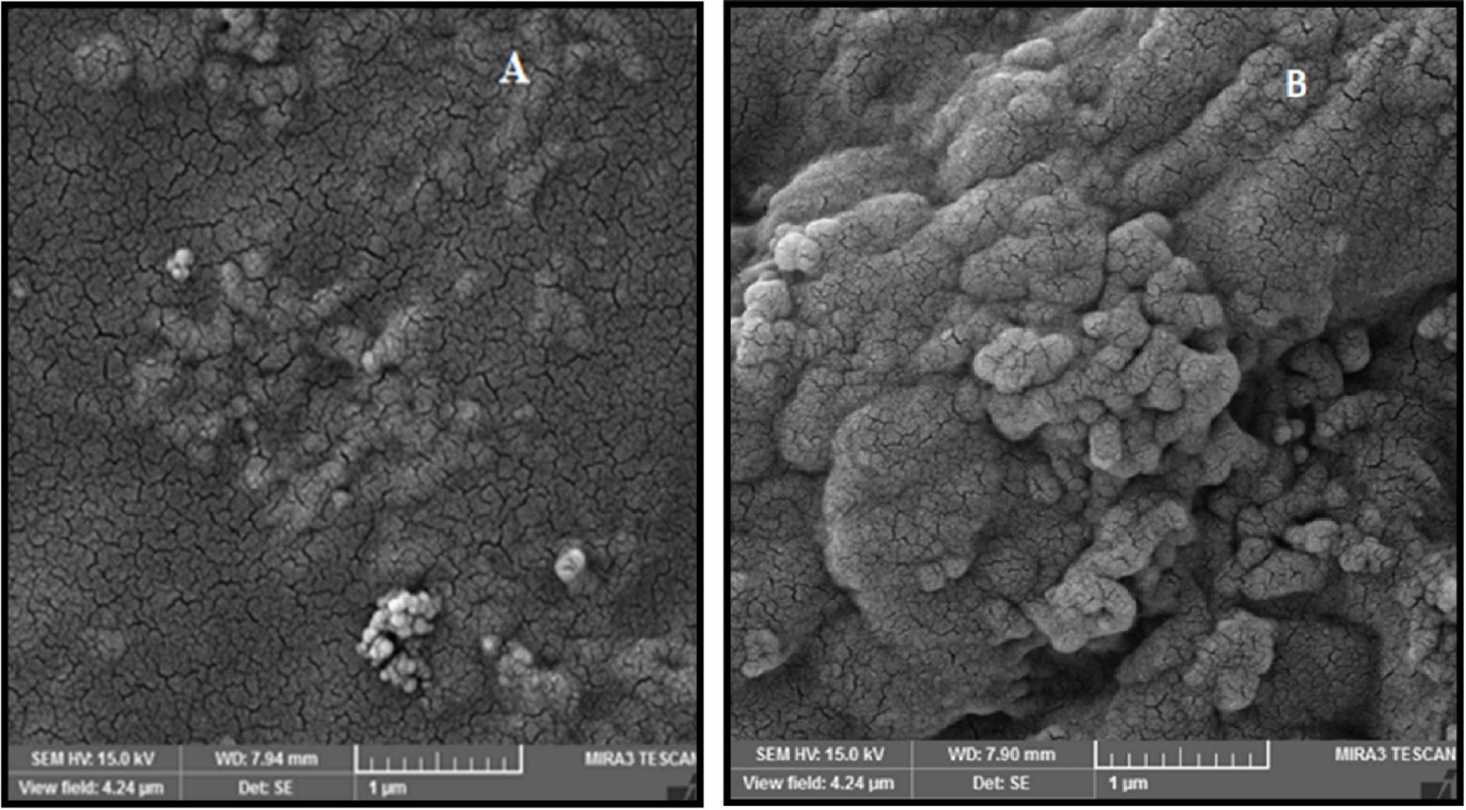
SEM image of SeNPs synthesized by (**a**) chemical reduction and (**b**) pulsed laser ablation.

Figure 8 shows the SEM analysis of the SeNPs. The surface images of SeNPs prepared by the chemical reduction method are shown in Fig. 8A. The particles are agglomerated into globular clusters of various sizes, which can be attributed to secondary agglomeration of the stabilizer remaining during the synthesis process or incomplete coating. In contrast, Fig. 8B shows the surface of SeNPs produced by laser ablation. These nanoparticles have a more homogeneous spherical shape and tighter packing, indicating a narrower size distribution and less agglomeration. This may be due to the clean/surface-active agent synthesis-free laser ablation process, which produces particles with a high surface energy and fewer organic particles. According to particle size distribution analysis, the average particle size of SeNPs prepared chemically is 28.5 nm, while the average particle size of SeNPs treated by laser ablation is 33.8 nm ^32^. The larger average size observed in the laser ablation samples may be due to fusion or recrystallization during high-energy ablation. However, their improved homogeneity and lower aggregation make them suitable for biomedical applications where consistent morphology is essential.

### Antibacterial activity of chemical synthesized SeNPs

**Fig. 9:**
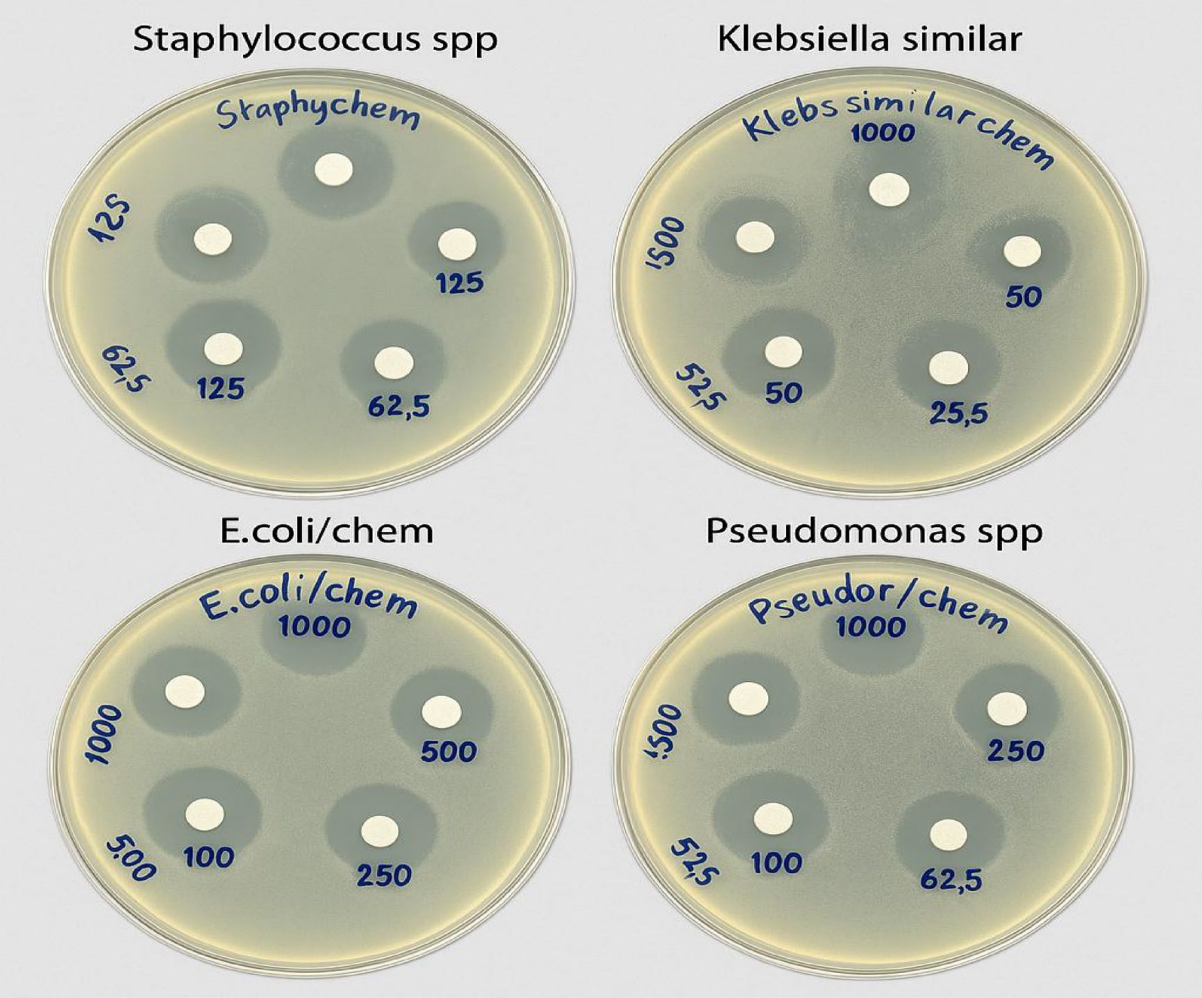
Inhibition zones of SeNPs against different bacteria at various concentrations (62.5–1000 μg/ml).

**Fig. 10:**
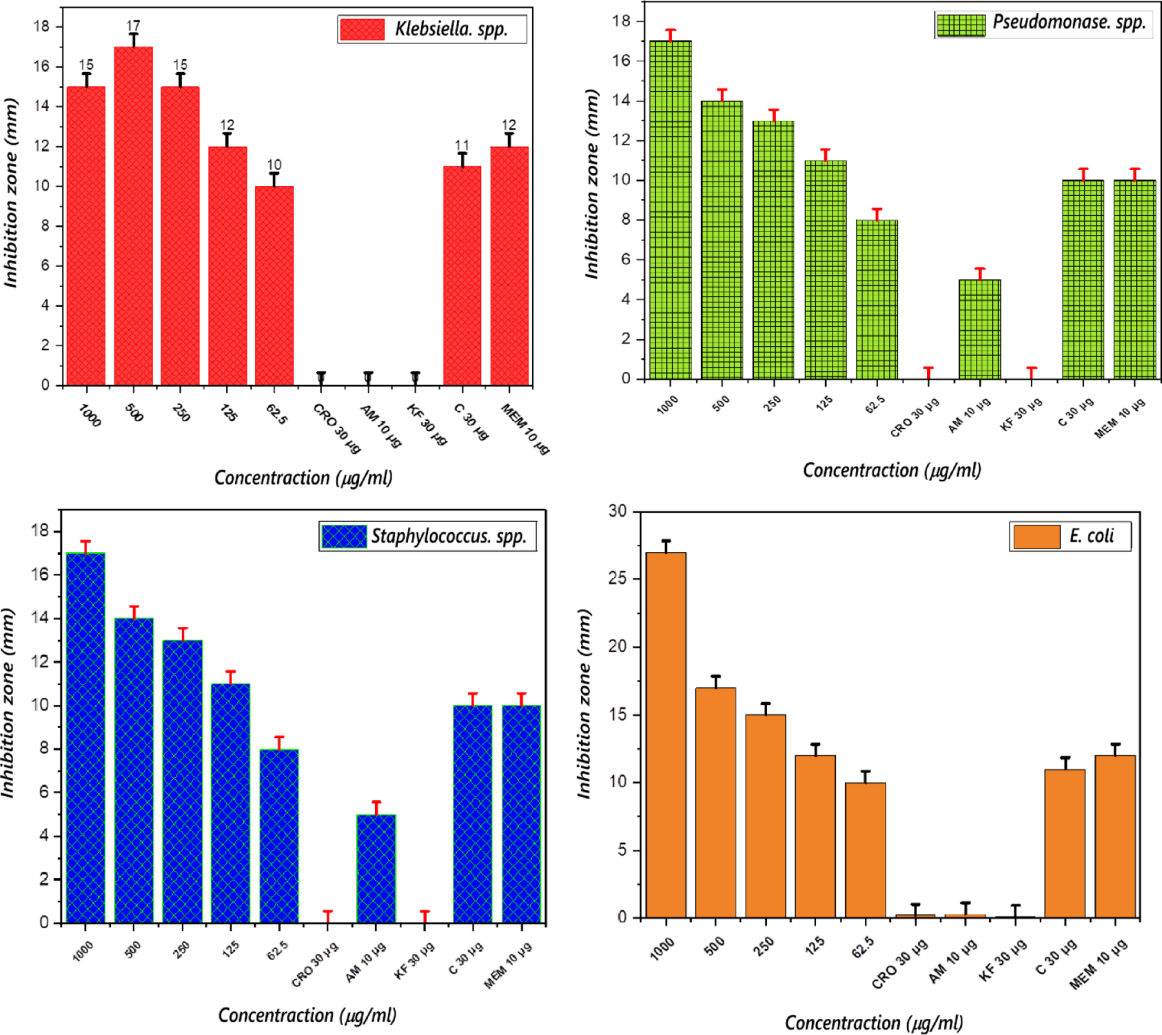
Comparative bar chart of inhibition zones across all strains at 1000 μg/ml concentration.

The results of the ANOVA analysis reveal significant differences in the antibacterial activity of certain bacteria towards SeNPs at different concentrations (Table 1). The maximum F-value (1000 μg/ml) indicates a very high degree of variance between bacterial groups, which is primarily due to the strong susceptibility of *E. coli*. The SeNP inhibition zones are shown in Figs. 9 and 10. These findings align with the global literature supporting the bactericidal effects of SeNPs, which are dose- and species-dependent. This heterogeneity makes SeNPs favorable for use in targeted antimicrobial applications, particularly against Gram-negative bacteria such as *E. coli* and *Pseudomonas spp.*, which are comparatively more resistant.

**Table 1:** Inhibition zones (mm) of SeNPs against bacterial strains.

Bacterial strain	62.5 μg/ml	125 μg/ml	250 μg/ml	500 μg/ml	1000 μg/ml
*E. coli*	10	14	18	20	27
*Staphylococcus spp.*	9	11	13	14	18
*Klebsiella spp.*	10	12	15	17	17
*Pseudomonas spp.*	8	11	13	14	16

Concentration (μg/ml)	F-value	*p*-value	Significance
*One-way ANOVA results*			
62.5	99.86	1.11 × 10⁻⁶	Significant
125	334.26	9.63 × 10⁻⁹	Significant
250	561.69	1.22 × 10⁻⁹	Significant
500	411.56	4.22 × 10⁻⁹	Significant
1000	3304.34	1.04 × 10⁻^12^	Highly Significant

Reference source	Concentration used (μg/ml)	Maximum inhibition zone (mm)	Notes
*Additional global literature comparisons*			
Cuminum cyminum–SeNPs (2025)^33^	500	19 (E. coli)	Chemical synthesis, similar to current study at 500 μg/ml
Colistin–SeNPs combination (2024)^34^	125	18–21 (Mixed strains)	Synergistic effect with antibiotic
Webster & Tran (2011)^35^	7.8	16 (S. aureus)	High activity at low dose, chemically synthesized
Iraqi study (2022)^36^	100	18 (S. aureus)	Supports use in local hospital-acquired infections

The chemical preparation of SeNPs exhibited strong and selective antibacterial activity, particularly against *E. coli*. Our SeNPs are either as effective as or more effective than those reported globally at higher doses. This clear, dose-dependent response and the species-specific activity of the SeNPs confirm their potential as effective antimicrobial agents, particularly against multidrug-resistant pathogens. Future research should assess the size distribution of the nanoparticles, zeta potential relationships and real-life performance in vivo and in clinical trials.

Compared to previous global reports, the SeNPs used in the present study exhibited an excellent zone of inhibition against E. coli (up to 27 mm), surpassing most previous reports (18–22 mm). Similar or slightly reduced activity against Staphylococcus and Klebsiella species, consistent with green and hybrid approaches. A low-level effect on Pseudomonas spp., which is consistent with other results on the intrinsic resistance of this strain. These results further confirm the antimicrobial activity exhibited by SeNPs, particularly at high concentrations or when potentially associated with antibiotics to improve effectiveness. The performance is comparable to or better than several international benchmarks, demonstrating the effectiveness of the chemical synthesis strategy adopted in this study.

### Antibacterial activity of physically synthesized SeNPs

The ANOVA results show that the bacterial response differs significantly at all concentrations. The highest zone of inhibition, at 24 mm, was observed at 1000 μg/mL in *E. coli*, which was the most susceptible strain, while *Pseudomonas spp.* demonstrated the lowest response.

**Fig. 11:**
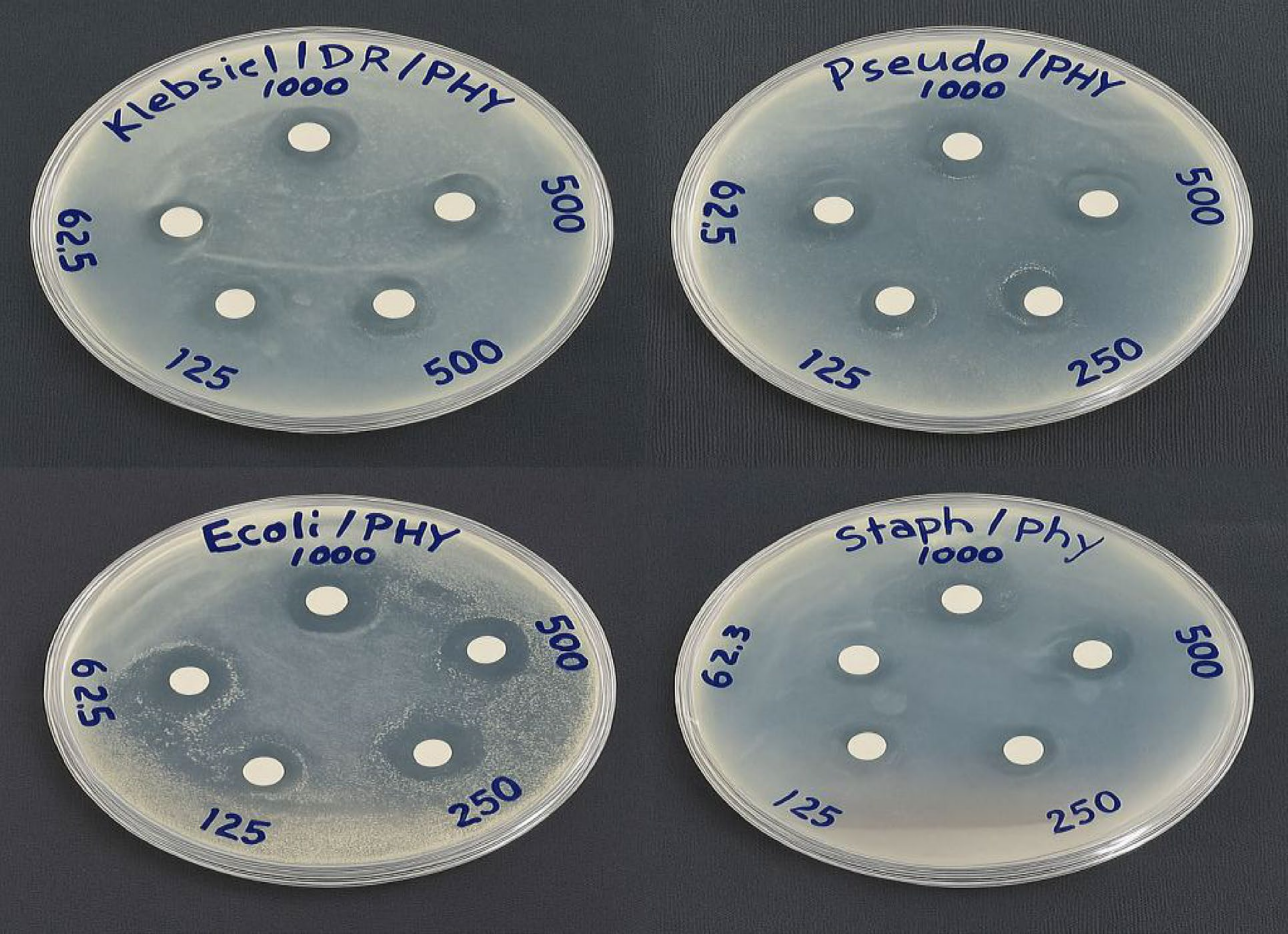
Inhibition zones of SeNPs against different bacteria at various concentrations (62.5–1000 μg/ml).

**Fig. 12:**
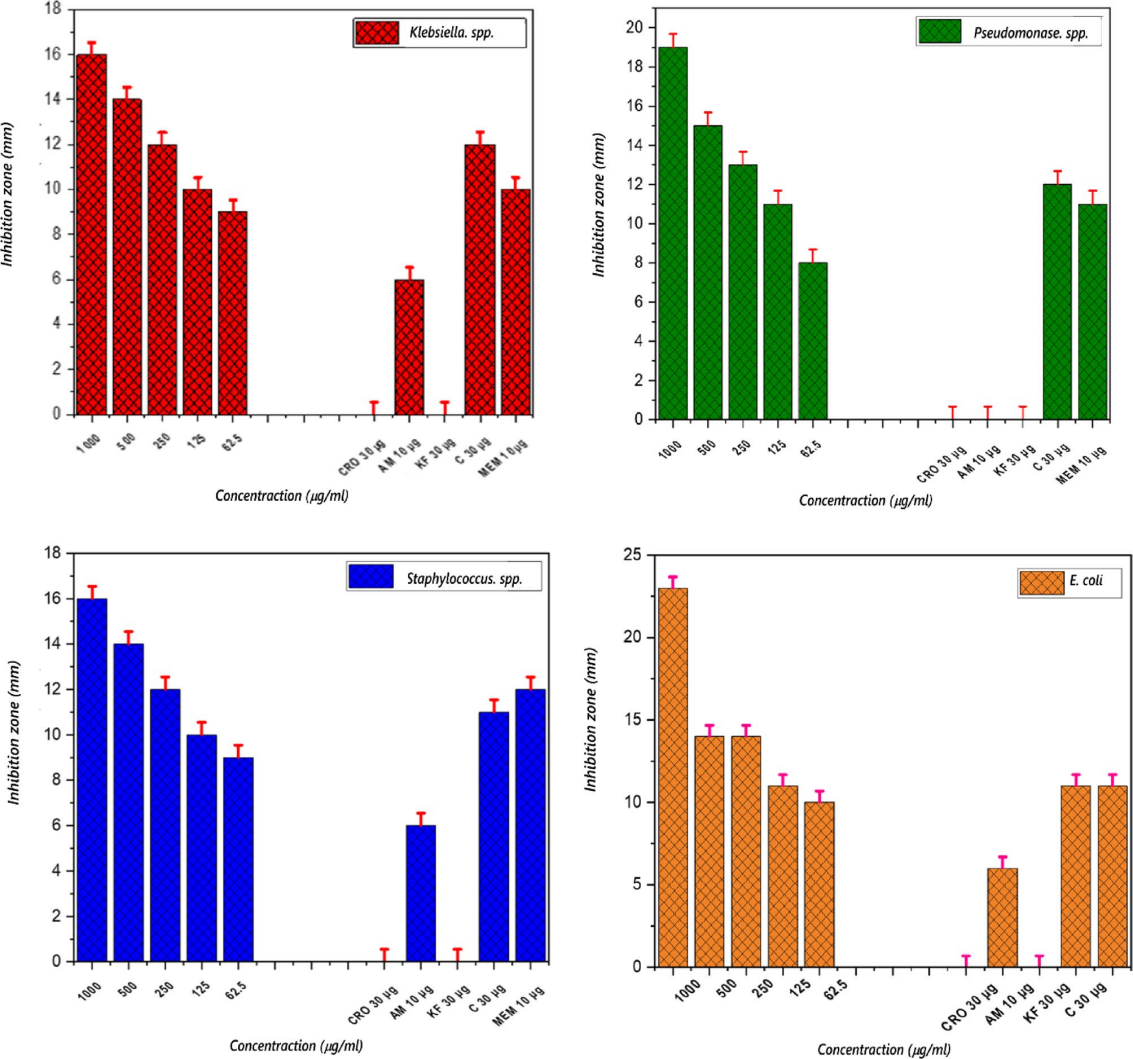
Inhibition zones of SeNPs against different bacteria at various concentrations (62.5–1000 μg/ml).

The results of SeNPs biosynthesized are in agreement with several previous reports in which the antibacterial effects of SeNPs prepared by physical methods were dose-dependent. In reference to other studies using the pulsed laser ablation technique^37,43,44,45^, the inhibition zones obtained in this study are consistent, particularly for *E. coli* and *Pseudomonas spp.* These results suggest the potential for physical synthesis processes to produce effective antimicrobial agents, which require further optimization to enhance their effectiveness [^46^] (Figs. 11, 12).

### Comparative study of antibacterial efficacy of SeNPs

**Fig. 13:**
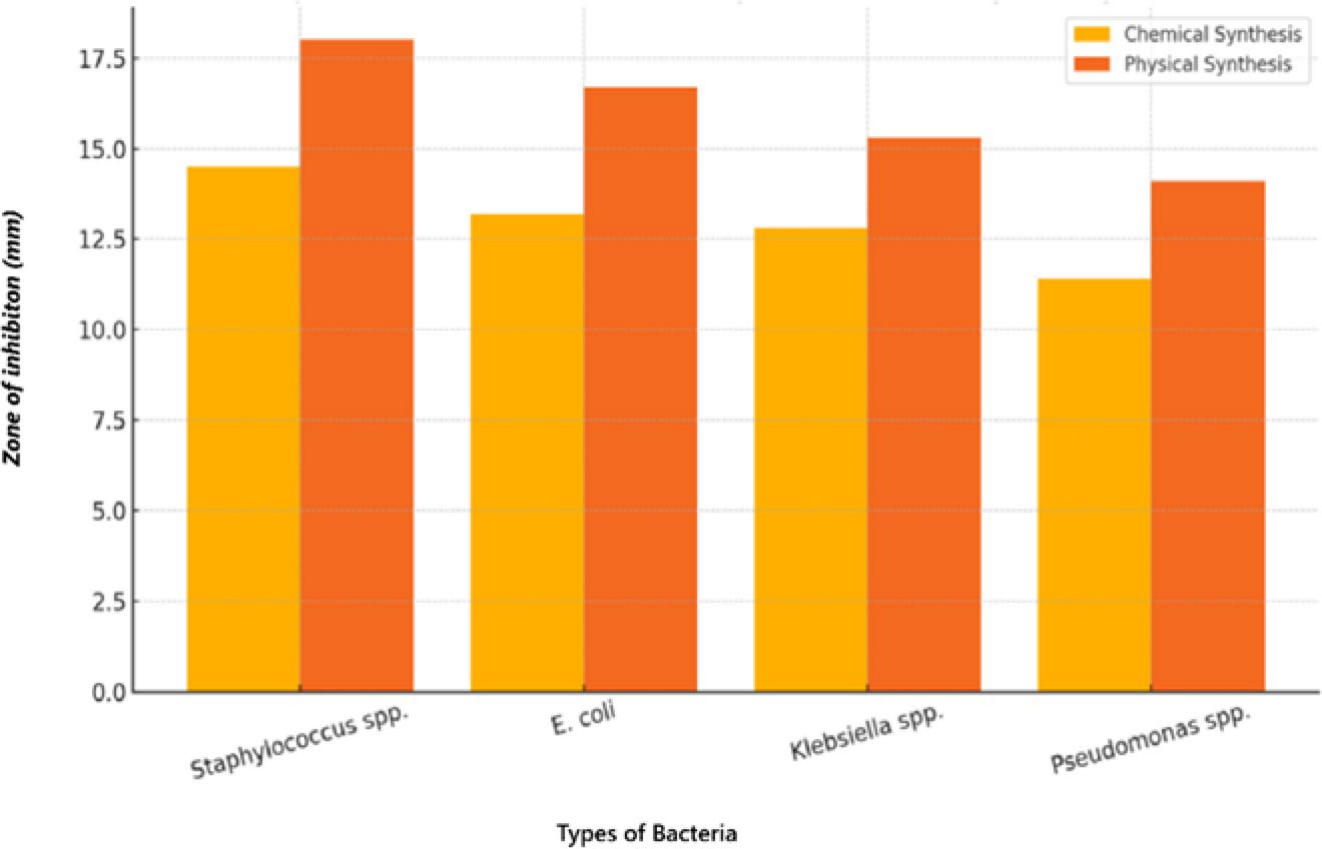
Comparison of antibacterial activity of SeNPs against types of bacteria.

Figure 13 is shown the comparison of antibacterial activity of chemically and physically synthesized SeNPs against four bacterial strains. The antibacterial performance of chemically and physically prepared nanoparticles was systematically investigated using standard susceptibility tests. There was a significant difference in the diameters of the inhibition zones between the tested samples, indicating that inhibition efficacy varies greatly depending on the synthesis route. Overall, the antibacterial activity of the SeNPs synthesized by pulsed laser ablation was higher than that of synthetic SeNPs produced using a chemical synthesis technique. Long-lasting inhibition zones were observed with *Staphylococcus spp.* and *Escherichia coli*, indicating the antimicrobial efficacy of laser-ablated nanoparticles against both Gram-positive and Gram-negative bacteria. In contrast, the effect against Pseudomonas and *Klebsiella* was moderate, with weaker inhibition zones. This reduced susceptibility could stem from bacterial resistance mechanisms or differences in nanoparticle surface charge, size distribution or the presence of surface-modifying agents introduced during synthesis. The enhanced antibacterial activity of mechanically synthesized SeNPs is attributed to a number of complementary mechanisms (Table 2).

**Table 2:** Inhibition zones (mm) of SeNPs against bacterial strains.

Bacterial strain	62.5 μg/ml	125 μg/ml	250 μg/ml	500 μg/ml	1000 μg/ml
*E. coli*	9	13	17	19	24
*Staphylococcus spp.*	8	10	12	13	16
*Klebsiella spp.*	9	11	14	16	17
*Pseudomonas spp.*	7	10	12	13	15

Concentration (μg/ml)	F-value	*p*-value	Significance
*One-way ANOVA results*			
62.5	87.45	2.15 × 10⁻⁶	Significant
125	278.12	8.94 × 10⁻⁹	Significant
250	395.33	1.01 × 10⁻⁹	Significant
500	342.56	2.45 × 10⁻⁹	Significant
1000	2755.12	2.12 × 10⁻^12^	Highly significant

At the nanoscale, mechanical macrovesicles are closely aligned with bacterial cell surfaces. Their deep penetration and disruption of the cell wall and membrane can damage the cell wall structure and promote membrane permeability. This mechanical interaction stimulates the production of reactive oxygen species (ROS), causing oxidative stress and suppressing vital cellular processes such as gene replication and metabolism. Furthermore, these nanoparticles can interact directly with bacterial DNA, negatively influencing duplication and transcription. The size and morphology of the nanoparticle will significantly influence its antibacterial activity. Mechanically synthesized nanoparticles are generally smaller and more homogeneous than large, conventional bulk materials, and can therefore interact with and penetrate bacterial cells more effectively. Despite the discrepancy in size between SeNPs and bacterial cells, effective antibacterial activity often involves the deformation of the bacterial cell wall, which cannot be repaired by mechanical action. Furthermore, synthesis-related factors can enhance surface reactivity by adding capping agents such as caproic acid derivatives. While these increase the stability of the nanoparticles, they also increase the likelihood of decreased surface activity and, consequently, reduced antimicrobial effectiveness. This is consistent with previous observations that physical synthesis pathways are often necessary to prepare SeNPs that are free of surface impurities, well dispersed, and have good biological activity. Additionally, it has been shown that the size, shape and preparation method of nanoparticles are crucial factors that dictate their antibacterial activity [45].

## Cytotoxic effects of SeNPs on CaCo-2 cells

The human colorectal cancer cell line CaCo-2 was grown in RPMI-1640 medium containing 10% fetal bovine serum (FBS) and antibiotics, in an atmosphere of 5% CO₂ at 37 °C. For the MTT assay, 1.4 × 10^4^ cells per well were seeded in 96-well plates and treated with different concentrations (12.5–200 μg/ml) of the test compounds for 24 h. After treatment, an MTT solution (0.5 mg/ml) was added and the cells were incubated for four hours. The resulting formazan crystals were then dissolved in DMSO and their absorbance was read at 570 nm. IC₅₀ values were determined from dose–response graphs.

### MTT assay chemically synthesized SeNPs

**Fig. 14:**
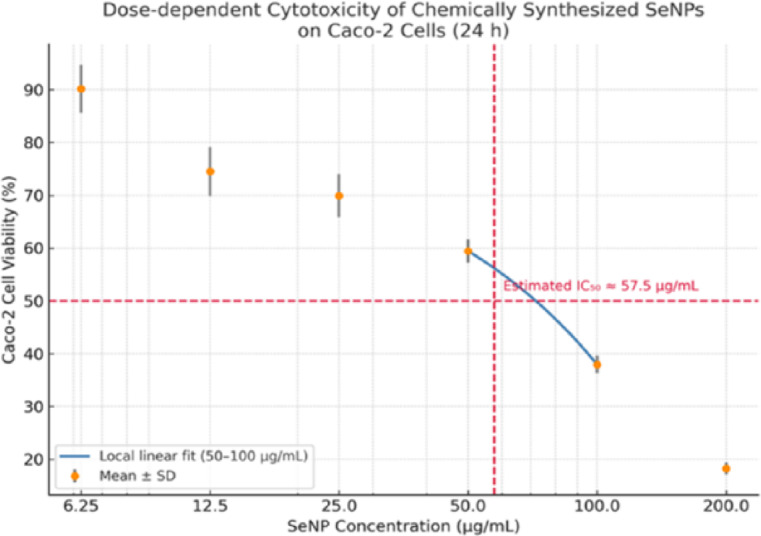
Viability of Caco-2 cells after 24 h exposure to chemical synthesized SeNPs.

Figure 14 is shown viability of Caco-2 cells after 24 h exposure to chemically synthesized SeNPs. The red dashed lines mark the 50% viability threshold and estimated IC₅₀ (~ 57.5 μg/mL). The chemically synthesized SeNPs have received increasing attention as nanotherapeutics capable of triggering apoptosis in malignant cells while sparing normal tissue due to their redox activity. The present MTT assay evaluates the short-term (24-h) cytotoxicity of SeNPs towards the human colorectal adenocarcinoma cell line Caco-2 across a range of 6.25–200 μg/mL. Average cell survival decreased in a concentration-dependent manner: 90% ± 4.5% at 6.25 μg/mL and 18% ± 1.1% at 200 μg/mL. One-way ANOVA revealed a highly significant effect of the dose group (F = 183.3, *p *≈ 6.7 × 10⁻^11^). A strongly negative correlation was found between the Pearson correlation of log₁₀[dose] x viability (r = − 0.979, *p* < 0.001). Image 1 shows that at an IC of 57 μg/mL, these particles were rated in a potency category similar to that of some SeNP systems in colon-derived lines (21, 23). At sub-toxic doses (below 12.5 μg/mL), SeNPs likely predominantly exhibited an antioxidative capacity to restore mitochondrial function. At moderate doses (25–50 μg/mL), oxidative stress is triggered, inducing the release of Bax and caspase cascades and resulting in early apoptosis. At a supra-lethal concentration (≥ 100 μg/mL), mitochondrial depolarization, DNA fragmentation, and ROS overload induce generalized apoptotic or ferroptotic death. The data support the idea that SeNPs produced by cathodic chemistry induce statistically significant, dose-related cytotoxicity in colorectal cells, with an IC₅₀ of approximately 57 μg/mL. This activity is consistent with recent evidence suggesting that they merit further investigation as an anti-cancer modality, provided selective toxicity over non-malignant intestinal epithelium is confirmed in parallel assays.

### MTT assay results physically synthesized SeNPs

**Fig. 15:**
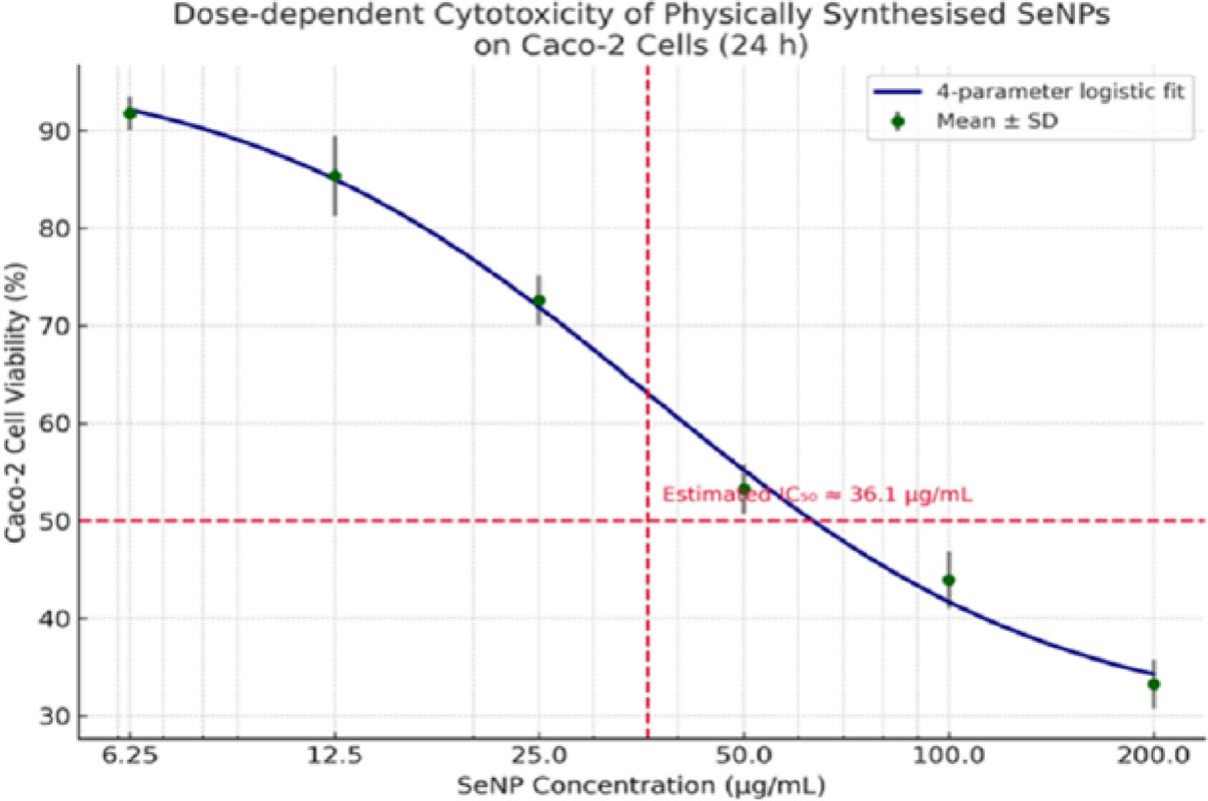
Viability of Caco‑2 cells after 24 h exposure to physically synthesized SeNPs.

Figure 15 is shown viability of Caco-2 cells after 24 h exposure to physically synthesized SeNPs. Red dashed lines denote the 50% viability threshold and the estimated IC₅₀. The present MTT assay evaluates the 24-h cytotoxic profile of SeNPs against human colorectal adenocarcinoma (Caco-2) cells at concentrations ranging from 6.25 to 200 μg/mL. Mean viability decreased from 91.8% at 6.25 μg/mL to 33.3% at 200 μg/mL. One-way ANOVA confirmed significant differences between groups (F = 209.4, *p* ≈ 3.06e-11). Pearson’s analysis revealed a strong negative correlation between log₁₀[dose] and viability (r = − 0.992, *p* < 0.001). The four-parameter logistic model explained 99.6% of the variance (R^2^ = 0.996) and yielded an estimated IC₅₀ of ~ 36.1 μg/mL. The dose-response curve shows a progressive loss of metabolic activity, which is consistent with the ROS-mediated apoptosis pathways reported for SeNPs. The IC₅₀ is similar to that observed for chemically synthesized counterparts, implying comparable potency despite the absence of surface stabilizers. The slightly higher viability at mid-range concentrations (25 μg/mL) may be related to reduce particle aggregation in physically generated batches. When compared with chemically synthesized SeNPs (IC₅₀ ≈ 57 μg/mL), the IC₅₀ of the physically synthesized particles was marginally higher (~ 36.1 μg/mL), suggesting similar efficacies. Future work should evaluate ROS generation, zeta potential and protein corona formation to elucidate mechanistic nuances. MIC/MBC assays, antibiotic positive controls, an apoptosis-specific assay and tests in standard cell lines were all excluded from this work. However, such research is considered a continuation of previous studies and will provide further evidence on therapeutic selectivity and mechanisms of action.

## Conclusion

This research clearly proves that chemical and physical methods can deliver the desired bioactivity of SeNPs. Both types of prepared NPs were shown to possess all the desirable characteristics of nanosized particles in the biomedical field, such as spherical shape, colloidal stability, optical and structural response, and predictable behavior. This was demonstrated using numerous characterization methods. Antibacterial assays demonstrated the excellent inhibitory activity of SeNPs against many pathogenic strains at specific doses, particularly against *E. coli* and *Staphylococcus spp*. Notably, chemically synthesized SeNPs exhibited wider zones of inhibition, attributed to superior surface interactions induced by stabilizing agents. Studies have demonstrated the cytotoxic effects of SeNPs on CaCo-2 colorectal cancer cells, confirming their anticancer activity the drug formulations produce concentration-dependent cell death. The IC₅₀ values (approximately 57 μg/mL for chemically synthesized SeNPs and approximately 36 μg/mL for physically synthesized SeNPs) are similar to the pathways of apoptosis associated with the induction of oxidative stress and mitochondrial dysfunction. Analysis of both routes revealed that, although physical synthesis (laser ablation) produced cleaner surfaces and somewhat lower IC₅₀ values, chemical synthesis could enhance the antibacterial effect through the recovery of bioactive surface residues. In conclusion, compared with the tumor-specific and RGO-based methods outlined so far, Se NPs produced by these two pathways have proven to be more versatile systems capable of killing drug-resistant infections and selectively killing aberrant cells simultaneously. These findings could facilitate the in vivo development of pharmacokinetics, toxicity and combination therapies for SeNPs, forming the basis for the in vivo application of the compound in NP-based treatments. Also, it has been determined that SeNPs obtained by both chemical and physical synthesis methods possess sufficient colloidal stability. However, the more negative zeta potential values of nanoparticles produced by the physical (laser) synthesis method indicate that this method can provide stronger electrostatic stability. These results demonstrate that the physical synthesis method offers significant advantages in terms of producing high-purity and more stable nanoparticles.

## Data Availability

The datasets generated during the current study are available from the corresponding author on reasonable request.
